# Differences between Dual Users and Switchers Center around Vaping Behavior and Its Experiences Rather than Beliefs and Attitudes

**DOI:** 10.3390/ijerph15010012

**Published:** 2017-12-23

**Authors:** Karolien Adriaens, Dinska Van Gucht, Frank Baeyens

**Affiliations:** 1 Faculty of Psychology and Educational Sciences, KU Leuven—University Leuven, Tiensestraat 102, 3000 Leuven, Belgium; dinska.vangucht@thomasmore.be (D.V.G.); frank.baeyens@kuleuven.be (F.B.); 2 Applied Psychology Unit, Thomas More University of Applied Sciences, Molenstraat 8, 2018 Antwerp, Belgium

**Keywords:** electronic cigarettes, dual use, tobacco harm reduction

## Abstract

(1) Background: Many smokers completely switch to vaping (switchers), whereas others use e-cigarettes (e-cigs) alongside tobacco cigarettes (dual users). To the extent that dual users substantially lower the number of cigarettes, they will reduce health risks from smoking. However, from a medical point of view, exclusive vaping is preferable to dual use; (2) Methods: Using an online questionnaire we assessed behavioral, cognitive and attitudinal aspects of e-cig use in smoking and ex-smoking vapers; (3) Results: Our sample consisted of 19% dual users and 81% switchers. Before e-cig initiation, both groups smoked on average 22 cigarettes per day (CPD). After e-cig initiation, dual users decreased tobacco consumption by 82% and were low-to-moderately cigarette dependent. Both groups had been vaping for on average 22 months, were highly e-cig dependent, used state-of-the-art e-cigs, nicotine concentrations of 4–8 mg/mL and often flavors other than tobacco. Dual users used substantially less e-liquid per week than switchers but reported a similar number of puffs/day, experienced less e-cig efficacy, more practical problems, more negative and less positive consequences, and endorsed smoking reduction (rather than quitting) as a more important reason to start vaping. For both groups, e-cig risk perception was low and little stigmatization was experienced. Dual users preferred tobacco cigarettes in stressful situations and when rapid nicotine uptake is required. E-cigs were preferred where cigarettes are prohibited and to reduce second-hand smoke; (4) Conclusions: Differences between dual users and switchers center around variables proximal to the vaping behavior and its experienced effects rather than hinging on more general vaping-related beliefs and attitudes.

## 1. Introduction

According to the most recent Eurobarometer regarding the attitudes of Europeans towards tobacco and electronic cigarettes, there has been a decrease in smoking prevalence followed by a stagnation in most European countries since 2006 [1]. In the EU, 26% of the population over 15 years old are smokers, of which most are daily smokers (24%). At the same time, both in the EU (43%) and in the U.S. (27%), substantial numbers of smokers report smoking less than 10 cigarettes per day (CPD) [1,2]. Even though this low-intensity smoking results in lower premature (all-cause and cause-specific) mortality risks than high-intensity smoking (more than 10 CPD), this should not take away from the fact that smoking one to 10 CPD (and even fewer than one CPD) is clearly associated with substantially higher premature mortality risks than never smoking [3]. The strongest positive associations with smoking-related causes of death, for smokers who consistently smoked less than one or one to 10 CPD, were observed for lung cancer (hazard ratio (HR) 9.12–11.61) and respiratory disease (HR 6.00). Likewise, results from the “One million women study” indicate that, while the risks of smoking increase monotonically with more cigarettes smoked, women who smoked less than 10 CPD also showed a substantially increased risk of mortality due to chronic lung diseases, lung cancer, coronary heart diseases, and stroke compared to never smokers [4]. They showed a doubling of the all-cause 12-year mortality rate of never smokers. In sum, low-intensity smoking for longer periods of time can also clearly have negative health outcomes [3,4].

Over the last few years, in many Western countries electronic cigarette (e-cig) use, a low-risk nicotine product often used as a form of tobacco harm reduction (THR), has (slowly) increased [1,5,6]. The most recent Eurobarometer (2017) showed that of all respondents (≥15 years old) in the EU, 15% had tried e-cigs, of whom 9% had just tried them once or twice (never regular use), 4% had regularly used them in the past but were not currently using them, and only 2% were currently using e-cigs regularly [1]. For the U.S., in 2016, 15% (≥18 years old) had tried e-cigs, of whom 3% were currently using them [7]. E-cig users (vapers) can be divided in two groups, namely those who are using regular tobacco cigarettes in combination with e-cigs (dual users) and those who use e-cigs exclusively, without smoking tobacco cigarettes. It is important to note that several studies indicate that regular e-cig use among never smokers is rare, so that most exclusive e-cig users are former smokers who quit smoking using an e-cig (switchers) [1,5,8,9]. The fact-sheet from ASH (Action on Smoking and Health) on current e-cig use by smoking status among adults in Great Britain showed that in 2014 63% of regular vapers were dual users and 35% were switchers, but more recent numbers show the opposite pattern of results (45% dual users and 52% switchers in 2017) [5]. In the U.S., in 2016 54% of current vapers were dual users and 34% were switchers [10].

Several surveys showed that most dual users reduce their amount to 10 or less CPD, implying that most of them become low-intensity smokers [5,9,11,12]. However, given the risks of low-intensity smoking, it is most likely that dual users are exposed to substantially higher health risks than switchers [3,9]. Therefore, from a health point of view, it is a non-trivial issue to understand why some vapers stay (or become) dual users and others become switchers. To the extent that a subgroup of dual users is preferring to switch completely but is currently failing to do so, this knowledge could help to assist dual users to quit smoking completely. The main focus of this study was to investigate differences between dual users and switchers in terms of behavior, cognitions and attitudes about e-cig use. Some of these differences can potentially point to elements to be addressed in interventions aiming at assisting dual users who want to quit smoking completely. 

To our knowledge, research directly comparing dual users and switchers is scarce. Rass, Pacek, Johnson and Johnson focused only on dual users by conducting a cross-sectional online survey in the U.S. to assess the characteristics, use patterns and harm perceptions of the e-cig and tobacco cigarette among dual users [13]. Dual use was described as using both products for at least three months and during the past week. The authors found that dual users smoked more frequently than using their e-cig (although there was a 30% decrease in the number of smoked CPD), had a higher dependence on their regular tobacco cigarettes than on vaping, showed little technical knowledge of their e-cigs, used e-cigs primarily because they believed they were less harmful, and mostly experienced vaping as less enjoyable than smoking tobacco cigarettes. Rass and his colleagues assessed the likelihood of using an e-cig or regular tobacco cigarette in different situations [13]. Dual users preferred to use their e-cig indoors, in restaurants, in the car, at the airport, around family in general and at work. In contrast, they preferred tobacco cigarettes when drinking alcohol, outdoors, when stressed or anxious, after eating, when drinking coffee, and after having sex [13]. These findings are largely similar to those found in a focus group study amongst dual users by Pokhrel, Herzog, Muranaka, Regmi, and Fagan: Tobacco cigarette use was more pronounced in situations of stress, when a strong craving for nicotine was experienced and in combination with other substances like coffee and alcohol [14]. On the other hand, e-cigs were used when e-cig use was perceived as more appropriate (e.g., inside vehicles, before/after physical activity) and as discrete alternatives in situations where tobacco cigarettes were prohibited or socially less accepted. 

Farsalinos and his colleagues conducted an online survey to assess the characteristics and experiences of over 19,000 e-cig users “worldwide” (75% e-cig consumers from Europe and 21% from the U.S.), including an assessment of differences between dual users and switchers with respect to demographics and smoking history, patterns of e-cig use and experienced benefits and side effects [9]. The sample consisted of dedicated e-cig users and 81% were switchers. The results showed that, before e-cig initiation, switchers had a higher dependence on tobacco cigarettes as measured with the Fagerström Test for Cigarette Dependence (FTCD) [15], had smoked longer and slightly more CPD and had undertaken more quit attempts in comparison to dual users. When dual users and switchers were matched for age and gender, differences in FTCD-scores and number of past quit attempts disappeared and switchers appeared to have been smoking less long but slightly more CPD [11]. At e-cig initiation, switchers were more likely to use a higher nicotine concentration (>20 mg/mL) than dual users (18 mg/mL), but at the time of the survey both groups used nicotine levels of 12 mg/mL [9]. These results were confirmed after matching [11]. Switchers experienced the most benefits and most of them reported an improved physical condition. For both dual users and switchers, the most important reasons for e-cig initiation included reducing or quitting smoking and reducing second-hand smoke [9]. The study with the matched groups showed that the main correlates of dual use included the use of less advanced e-cigs, starting with lower nicotine levels at e-cig initiation, using e-cigs more occasionally (instead of daily), starting with e-cigs to avoid smoking bans in public places and the perception that e-cigs are equally or even more harmful than smoking [11].

We conducted an online survey and focused on those elements that potentially could point to elements to be included in an intervention aiming at dual users who want to quit smoking completely in the future. Our questionnaire was more comprehensive in comparison to those used in the previously discussed studies in that it also included perception of e-cig efficacy, practical problems with the e-cig, the social component of vaping, and the “addictive” component of smoking/vaping/nicotine.

## 2. Materials and Methods

### 2.1. Participants

To reach a sample of dual users and switchers, we distributed a link to an online Dutch questionnaire through social media (Facebook and Twitter) and a Dutch forum for vapers (www.dampforum.nu) in February and March 2016. In one month, 252 participants started filling out the questionnaire, with a total of 217 completing it. When analyzing the data, some logical inconsistencies in the pattern of answers made clear that 14 participants interpreted some questions differently than intended. We contacted those participants by e-mail for clarification and, eventually, to correct some of the answers. Only 12 of these 14 participants could be reached, meaning that the other two participants had to be excluded from the sample. Consequently, a total of 215 participants were included, consisting of 40 dual users (19%) and 175 switchers (81%).

Inclusion criteria were the following: being above the age of 18 and being a current dual user (that is, currently smoking and vaping) or a current switcher (that is, being an ex-smoker currently vaping). Smokers who currently exclusively smoked tobacco cigarettes could not participate. The mean age of participants was 43 years with the youngest being 18 and the oldest 73 (see Table 1). Most of the participants were Caucasian, had at least a high school or bachelor’s degree, worked full-time and were married or cohabiting.

### 2.2. Measures

The online questionnaire, made in Qualtrics [16], started with assessing several demographics (all predefined categories, with the option to provide additional information, see Table 1): age, gender, ethnicity, marital status, highest educational degree, occupation and monthly net income. Secondly, all participants were questioned on their smoking behavior of regular tobacco cigarettes, including (see Table 2): age when they started smoking (open ended), smoking behavior before e-cig initiation (product use, frequency of use, mean CPD), current smoking behavior (product use, frequency of use, mean CPD), quit smoking attempts (including the number of quit attempts and products used during these and the longest quit attempts), time since last cigarette smoked and current stop smoking motivation (predefined categories). 

In the third part of the questionnaire, participants were asked to fill out questions on their e-cig use (see Table 3). The following items were assessed: product use, nicotine concentration (mg/mL), average volume of e-liquid used per week (in mL), used flavors now and at e-cig initiation (tobacco or others), time since e-cig initiation (in months), current e-cig use (frequency of use, number of puffs/day), future plans concerning e-cig use and nicotine level, smoking/vaping behavior at time of e-cig initiation (initial dual use or initial immediate switch) and reasons (using Likert scales, see further) to start and to continue vaping. 

The fourth part of the questionnaire consisted of several statements concerning behavioral, cognitive and attitudinal aspects of vaping/smoking, to which participants could express their level of (dis)agreement using Likert scales ranging from 1 (“totally not agree”) to 5 (“totally agree”) (see Table 4). The different aspects questioned included: risk perception (perceived harmfulness of e-cig in absolute terms and in comparison to tobacco cigarette), e-cig efficacy (perceived efficacy of e-cig for craving reduction and as a cessation tool), practical problems (experienced practical problems when using an e-cig), a social component (restrictions imposed and stigmatization by the close environment), negative consequences (experienced negative consequences of e-cig use), positive consequences (experienced positive consequences of e-cig use), cigarette “addiction” (perceived “addiction” to cigarette smoking), e-cig “addiction” (perceived “addiction” to vaping), nicotine “addiction” (perceived “addiction” to nicotine). 

The fifth part of the questionnaire was only filled out by the dual users because it presented several situations in which dual users were asked to indicate if they preferred to use a tobacco cigarette or an e-cig. The questions were rated on Likert scales going from 1 (“certainly a tobacco cigarette”) to 5 (“certainly an e-cig”). The sixth and final part included measurements for tobacco cigarette dependence (only dual users) and e-cig dependence (dual users and switchers) using the FTCD and an adapted version for e-cigs [15].

### 2.3. Procedure

In January 2016, the study protocol was approved by the Social and Societal Ethics Committee (SMEC; KU Leuven, Belgium) (file number: G-2016 01 454). The questionnaire was online from February 8th until March 8th 2016. Interested people were first given the opportunity to read the informed consent. After giving their consent, they could fill out the questionnaire, which took 15 to 18 min. As a remuneration, participants had a chance to win one out of two vouchers of 25 euros for a local department store (FNAC, selling books, CDs, DVDs, computer equipment, etc.).

### 2.4. Statistical Analyses

Results (means; standard deviations, *SD*s; proportions) were computed for both the total sample as for the dual users and switchers separately. To compare dual users with switchers, variables were analyzed using Pearson’s chi square tests (categorical variables) for equal proportions or Fisher’s exact tests (when the expected n was below five in more than 20% of the cells) and independent *t*-tests (continuous variables) assuming equal variance. In general, alpha levels of 0.05 were used. However, in order to minimize type I errors, for the individual statements of the “reasons to start/continue vaping” clusters as well as for the items of the “behavioral/cognitive/attitudinal aspects” scales, alpha levels were adjusted familywise using the Bonferroni correction (alpha/*n* where *n* is the number of items in a given subset of items or scale). For these individual statements purportedly measuring one particular behavioral/cognitive/attitudinal aspect (see Measures section), sum scores were also calculated and compared between groups. These scales were tested on internal consistency using Cronbach’s alpha. All analyses were performed using SPSS, version 24.0 [17].

## 3. Results

### 3.1. Demographics

Dual users did not differ from switchers, all *p*’s > 0.08, on most general demographics (see Table 1), except for gender and age. Compared to the dual users, among the switchers there were more men than women, χ^2^ (1) = 7.90, *p* < 0.01, and switchers were slightly older, *t*(213) = −2.09, *p* < 0.05.

### 3.2. Smoking Behavior

Most smoking behaviors before e-cig initiation did not reliably differ between the two groups, all *p*’s > 0.05 unless indicated otherwise (see Table 2). Both dual users and switchers started smoking around the age of 15, they smoked daily, on average 22 CPD, and mostly filtered cigarettes or rolling tobacco. Almost 90% of both groups had tried to quit smoking in the past, but dual users reported more quit smoking attempts compared to switchers, *t*(188) = 3.21, *p* < 0.001. The longest period of having quit smoking, for both dual users and switchers, lasted on average 13 months.

An obvious difference was observed between dual users and switchers concerning the time since the last cigarette smoked, namely most dual users smoked their last cigarette a day or less ago (68%) and for most switchers this was longer than a year ago (55%), *p* < 0.001. Of those who had tried to quit smoking in the past, for both dual users and switchers, the most widely used cessation aids for quitting smoking were willpower (58%) and nicotine replacement therapy (NRT; 52%). The most widely used cessation aids for the longest quit smoking period, for both groups, were e-cigs (61%), and willpower (27%).

At the time of the survey, the switchers, as per definition, no longer smoked tobacco cigarettes and the dual users had decreased their average smoked CPD by about 82% from their pre-vaping CPD. Dual users were low to moderately tobacco cigarette dependent (*M* FTCD-score = 3). Half of the dual users were still smoking daily and 30% on multiple days per week (but not daily). Most of the dual users (83%) were smoking filtered cigarettes. One third of the dual users did not plan to quit smoking within the next six months, one third thought about quitting within the following six months and one third wanted to quit within the next month.

### 3.3. E-cig use

E-cig use characteristics did not substantially differ between the dual users and switchers, all *p*’s > 0.10 unless indicated otherwise (see Table 3). The two groups reported a very high dependence on e-cigs. On average, for both dual users and switchers, participants started vaping 22 months ago. They were mostly using state-of-the-art open system devices without modifications, liquid with a nicotine level between 4 and 8 mg/mL and a flavor other than tobacco. The latter is in contrast with the flavor they used at e-cig initiation, which was mainly a tobacco flavor. No differences were observed regarding e-liquid Propylene Glycol/Vegetal Glycerin (PG/VG) ratios: 37% used a 50/50 mixture, 27% used mainly propylene glycol and 36% used mainly vegetable glycerin. Both groups were using their e-cig daily. The puffing frequencies of dual users and switchers did not differ, but at the same time dual users consumed significantly less (about half) e-liquid than switchers, *t*(213) = −3.98, *p* < 0.001. For most of the dual users (65%) and switchers (67%), their future plans with e-cigs were to continue to use it as they were doing now. Concerning nicotine concentrations, 27% of all participants wanted to continue using the same concentration as they currently did, 32% wanted to decrease the nicotine concentration and 41% wanted to switch to zero nicotine.

We also questioned participants regarding how they had arrived at currently being a dual user or a switcher, namely, if they had initiated as a dual user or as an immediate switcher. For current dual users the probability that they had started as a dual user (0.70) was higher than the probability that they had started as an immediate switcher (0.30). For current switchers, on the other hand, the probability to have started as an immediate switcher (0.66) was higher than the probability to have started as a dual user (0.34). The probabilities differed significantly, χ^2^ (1) = 17.18, *p* < 0.001.

The main reasons for both dual users and switchers to start vaping (all *p*’s > 0.03; adjusted alpha level of 0.006) were that smoking is unhealthy, that vaping has more advantages compared to other cessation aids, and to quit smoking completely (see Table 3). Dual users agreed more than switchers with the statement that they had started vaping to reduce smoking, *t*(213) = 4.33, *p* < 0.001. Regarding reasons to continue vaping (all *p*’s > 0.09; adjusted alpha level of 0.007), the most important reasons for both groups were that smoking is unhealthy, that vaping has more advantages compared to other cessation aids and that vaping is pleasant. In addition to these shared reasons, dual users continued vaping because it helps to smoke less and to quit smoking completely in the future. For switchers, an important additional reason to continue vaping was that vaping prevents relapsing to smoking.

### 3.4. Behavioral, Cognitive and Attitudinal Aspects

For each of the main behavioral, cognitive and attitudinal aspects, a mean score was computed for the constituting items, see Table 4. Responses to all statements were measured/expressed on a five-point Likert scale ranging from “totally not agree” to “totally agree”. Most aspects showed an internal consistency of at least 0.80, only for “addiction” for cigarettes (0.73), the social component (0.65), e-cig efficacy (0.58), and “addiction” to nicotine (0.54) the consistency was lower than 0.80, but still in an acceptable range. Removing items did not increase the Cronbach’s alphas.

With respect to the risk perception of e-cigs, both groups (totally) disagreed with each of the statements expressing substantial health risks of vaping (all *p*’s > 0.21; adjusted alpha level of 0.007). Participants in both groups also perceived e-cigs as less harmful than tobacco cigarettes (all *M*’s < 2.63). Although both groups (strongly) agreed with the statements expressing e-cig efficacy, switchers endorsed slightly more e-cig efficacy than dual users, *t*(213) = −5.18, *p* < 0.001. Switchers agreed more than dual users with the items “*Less desire for cigarettes*”, “*After vaping I do not immediately smoke*”, “*No need for cigarette after vaping for a while*” and “*Throat hit is comparable to smoking*”, all *p*’s < 0.002 with adjusted alpha level of 0.007. Dual users experienced more practical problems than switchers, *t*(213) = 2.94, *p* < 0.01, although in absolute terms both groups indicated few problems (all *M*’s < 2.48). Dual users agreed more with the statements “*Battery is empty at inconvenient moments*” and “*Inconvenient to carry material of e-cig*”, all *p*’s < 0.001 with adjusted alpha level of 0.007. Dual users and switchers did not differ in their experiences of social stigmatization and social forces working against vaping. Both groups scored between neutral and agreeing on most items of the social dimension (most *M*’s > 3.29, all *p*’s > 0.06 with adjusted alpha level of 0.008), implying some experienced stigma and other social obstacles to vaping.

Although both groups (strongly) disagreed with most statements expressing experienced negative consequences of using e-cigs (all *M*’s < 2.87), dual users expressed slightly more negative consequences, *t*(213) = 2.92, *p* = 0.004. The statements on which dual users scored higher than switchers were “*Coughing*”, “*Bad taste when vaping*”, “*Bad physical condition*” and “*Sleeping problems*”, all *p*’s < 0.002 with adjusted alpha level of 0.003. In a similar vein, although both groups (strongly) agreed with most items expressing experienced positive consequences of vaping, switchers were more positive than dual users, *t*(213) = −5.45, *p* < 0.001, and agreed stronger with the following items: “*Craving for cigarettes reduced*”, “*By using the e-cig I could quit smoking*”, “*Smell/taste of cigarettes is less pleasant*”, “*Fitness and health are improved*”, “*Better breathing*”, “*Improved sense of smell*”, “*Improved taste*”, and “*Improved appetite*”, all *p*’s < 0.001 with adjusted alpha level of 0.003.

The final aspect concerns the perceived “addiction” to cigarettes, e-cigs and nicotine. Dual users agreed more than switchers with the statements expressing “addiction” to smoking, *t*(213) = 13.85, *p* < 0.001. This was expressed on the items “*I cannot resist the act of smoking*”, “*Addicted to cigarettes at this moment*” (all *p*’s < 0.001 with adjusted alpha level of 0.025) and they also saw their “addiction” to cigarettes as problematic. Perceived “addiction” to e-cigs did not differ between the two groups. Both groups were neutral to agreeing on the items expressing “vaping addiction” (adjusted alpha level of 0.025). Finally, both groups were neutral on “addiction” to nicotine, but on the specific item “*I feel addicted to nicotine*”, dual users agreed more than switchers, *t*(213) = 1., *p* = 0.02 (adjusted alpha level of 0.025).

### 3.5. Situations

**Figure 1:**
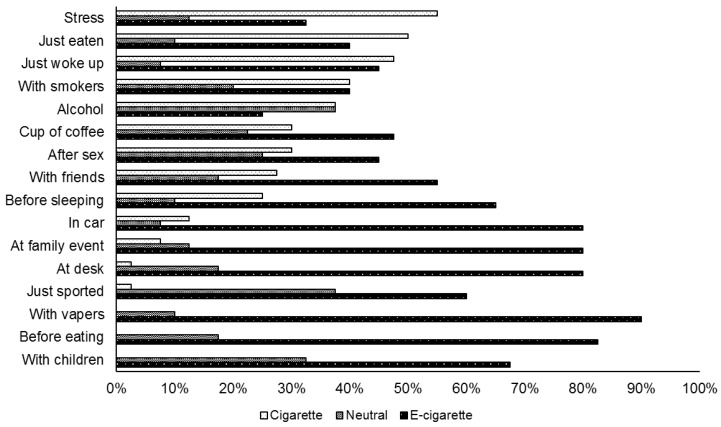
Situations in which dual users prefer cigarettes vs. e-cigs.

Dual users were more inclined to use their regular tobacco cigarettes in stressful situations, after eating, when waking up, around other smokers and when consuming alcohol, see Figure 1. In contrast, e-cigs were more preferred in the car, at family events, after playing sports, at the office, around children, before eating and around other vapers.

## 4. Discussion

The sample reached in this study consisted of 18% dual users and 81% switchers. These numbers are similar to the proportions of dual users and switchers obtained in some other web-based convenience-sample surveys: for example, in the “worldwide” survey by Farsalinos and his colleagues and in the recent study by Lehmann and her colleagues regarding e-cig users in Germany, the proportion of switchers was also found to outweigh the proportion of dual users by far [9,12]. However, this distribution is in contrast with results from representative population samples: for example, in the 2017 ASH survey, 45% of the vapers were found to be dual users and 52% were switchers [5], whereas in the 2017 Society against Cancer survey, 62% of Belgian vapers were dual users and 39% switchers [18]. Hence, the high prevalence of switchers in the current study is most likely due to the recruitment process. Our survey was distributed via social media and a Dutch forum for vapers such that it is highly likely that more dedicated vapers with success experiences with vaping filled out the questionnaire, resulting in more switchers than dual users.

Smoking behavior before e-cig initiation did not differ between dual users and switchers. The only exception was the number of past quit smoking attempts: dual users had made more attempts than switchers, which is the opposite pattern to the Farsalinos study [9]. After e-cig initiation, dual users decreased their cigarette consumption by around 82%, going from 22 to four CPD. Such a substantial reduction in CPD appears to be not unusual among dual users [9,11,12,19]. Moreover, the longitudinal study of regular vapers by Etter also showed that many dual users are able to quit smoking completely over time [20]. In our sample, from the 175 current switchers, 34% had started initially as a dual user, indicating that, at least for some, dual use may indeed be an intermediary step on a trajectory towards becoming a full switcher.

Our sample consisted of experienced vapers, vaping daily for around 22 months, using state-of-the-art e-cigs, vaping mostly non-tobacco flavors, and using nicotine concentrations of 4–8 mg/mL. Dual users were using significantly less liquid per week than switchers, although their puff frequency was the same. Farsalinos and his colleagues obtained similar results for dual users who were using less liquid compared to switchers [11]. Lehmann and her colleagues also observed this phenomenon, but this was partially compensated by (and attributed to) the higher nicotine concentrations that dual users were using compared to switchers [12]. One possible explanation for the current observation that dual users reported a similar puff frequency but consumed no more than about half the volume of e-liquid of switchers, might be that dual users take shorter puffs and/or vape at lower power settings and thus use less liquid than switchers. Consuming less e-liquid with a similar nicotine concentration inevitably results in the consumption of less nicotine. This in turn may explain—at least partially—why dual users still smoke some cigarettes, namely to compensate for the lower nicotine uptake with the complementary nicotine of smoking cigarettes. In this context, it is at least remarkable that a majority of both dual users and switchers wanted to continue vaping in the future, but also wanted to decrease nicotine concentrations and/or use nicotine free e-liquid.

Although—in switchers and dual users alike—the main reported reasons to start vaping were “*that smoking is unhealthy*”, “*that vaping has more advantages than other cessation aids*” and “*to quit smoking completely*”, dual users also agreed (and more so than switchers) with the statement that they had started vaping to reduce smoking. Farsalinos and his colleagues reported more differences between both groups, namely that dual users found economic reasons and avoiding smoking bans more important as a motivation to start vaping [11], whereas switchers reported reducing the health risks of smoking and reducing second-hand-smoking as more important. Lehmann on the other hand observed that the main reason for vaping initiation for dual users was to reduce, instead of to quit smoking [12].

Both switchers and dual users showed a low risk perception concerning e-cig use and perceived e-cigs as less harmful than cigarettes. This is in line with the study of Farsalinos and his colleagues [11]. In the study of Rass and her colleagues, however, there was more doubt among dual users if e-cigs are less harmful than tobacco cigarettes [13]. Next, we observed that in both groups e-cigs were perceived as efficacious and only a few practical problems were reported. However, dual users experienced slightly less efficacy and slightly more technical or practical problems compared to switchers. Restrictions imposed and stigmatization by the close social environment were not really experienced, but most vapers reported that they are expected to vape outdoors. Both groups experienced little negative and a lot of positive consequences from e-cig use, although dual users experienced slightly more negative and slightly less positive consequences than switchers. It is unclear whether the lower net experienced benefits of vaping in dual users are a cause or a consequence of the dual use: lower experienced benefits may lead some vapers to complementary smoking, or alternatively, the continuation of smoking (albeit limited) may lower experienced positive and enhance experienced negative effects of vaping [3,9]. Finally, whereas experienced “addiction” to e-cigs was the same for both groups, experienced “addiction” to tobacco cigarettes was more pronounced for dual users and they also felt more nicotine dependent compared to switchers. Again, it is unclear whether the latter differences should be seen as a cause or as a consequence of the continuation of smoking.

Lastly, our study reconfirmed previous research results regarding the situations where tobacco cigarettes or e-cigs are preferred [13,14] Dual users preferred smoking tobacco cigarettes in stressful situations and at moments when a rapid nicotine uptake is probably required (e.g., after wakening). E-cigs were rather preferred indoors and in situations with a higher risk of exposing others to second-hand smoke (e.g., family events).

All results and conclusions made must be seen in the light of the following limitations. First, the sample size reached is rather small. Second, the number of switchers outweighed the number of dual users by far. As noted earlier, it is highly likely that especially the “real enthusiast” and more dedicated vapers are active on social media and fora for vapers, and that those having success experiences with vaping were more willing to participate in our study, resulting in more switchers in our sample than in the general population of vapers. This (highly probable) self-selection bias implies that conclusions cannot be generalized to the overall population. Thirdly and lastly, causal conclusions regarding experienced benefits/complaints due to vaping/smoking cannot be made, due to the cross-sectional nature of the study design.

## 5. Conclusions

Overall, the results of the current study indicate that differences between dual users and switchers center around variables proximal to the vaping behavior (e.g., liquid consumption, e-cig efficacy, practical problems) and its experienced effects (positive/negative consequences) rather than hinging on more general vaping-related beliefs and attitudes (e.g., e-cig harm perception, social aspects of vaping), demographics, or smoking history. To some extent, reasons to start vaping (e.g., to reduce or quit smoking), e-cig initiation patterns (dual use/switching at start) and experienced “addiction” to smoking and nicotine might also be involved.

Some of these observed differences between dual users and switchers point to elements that could be addressed in interventions aiming at assisting dual users who want to quit smoking completely (see for example Meltzer and colleagues) [21], including the amount of e-liquid used, the reported practical problems, and the experienced lower e-cig efficacy. More specifically, it may be helpful to inform them on how to use more performant and efficacious e-cigs (e.g., state-of-the art open system e-cigs with variable wattage and high-quality coils) in a more efficient manner (e.g., taking longer puffs), on how to minimize practical problems (e.g., using a high-capacity battery and a sufficiently large tank), and on how to adapt e-liquid consumption and nicotine concentrations to their personal needs, especially in those situations in which dual users hitherto keep smoking tobacco cigarettes (e.g., consuming more liquid/day, with higher nicotine concentrations, and not progressing too quickly to low(er) nicotine concentrations). 

It remains equally important, however, to recognize and respect that not all dual users express the desire to quit smoking completely (in this sample only one third expressed the clear intention to quit smoking within the next month). Relatedly, some smokers who have tried vaping report that e-cigs are lacking some of the unique sensory-hedonic characteristics of cigarette smoking that they highly value and enjoy (e.g., the smell and flavor of burned tobacco), whereas at the same time vaping has some intrinsic characteristics that they dislike (e.g., the more “artificial” vaping experience, the complex technology, the “chemical nature” of e-liquids) [22]. To the extent that similar likes and dislikes also play a role in dual users’ choice to continue smoking, one cannot but acknowledge that e-cigs have their limits as a tool for THR, and welcome present or future other low-risk nicotine products that better address the hedonics of smoking.

## Figures and Tables

**Table 1:** Sociodemographic variables.

Variable	Total	Dual user	Switcher	Statistic	*p*-value
*Age ^a^*	*42.57 (11.23)*	*39.25 (13.08)*	*43.33 (10.66)*	*t(213) = −2.09*	*0.04*
*Gender*				*χ^2^ (1) = 7.90*	*0.005*
Man	75	58	79		
Woman	25	43	21		
Ethnicity					0.12 ^b^
Caucasian	98	95	98		
Asian	0	3	0		
Mix ethnicity	2	3	2		
Marital status					0.10 ^b^
Single/unmarried	20	35	17		
Married	42	30	45		
Cohabiting	31	28	32		
Divorced	5	5	5		
Widow(er)	2	3	2		
Highest degree					0.62 ^b^
Primary school	3	8	3		
High school	47	45	47		
Bachelor’s	40	38	40		
University	9	10	9		
Other	0	0	1		
Occupation					0.08 ^b^
Student	3	8	2		
Working part-time	15	20	14		
Working full-time	62	48	66		
Housewife/-man	8	5	9		
Job seeker	7	13	6		
Retired	5	8	4		
Monthly net income				χ^2^ (5) = 8.43	0.13
Under 1000 €	13	20	11		
1000–1500 €	15	15	16		
1500–2000 €	29	38	27		
2000–2500 €	22	8	25		
2500–3000 €	11	13	10		
More than 3000 €	10	8	10		

*Note*: all percentages (rounded to the nearest integer) except for ^a^, ^a^ are *M* (*SD*). ^b^ More than 20% of the expected frequencies less than 5, Fisher’s exact test was used. *p*-values in *Italic* are significant with α = 0.05.

**Table 2:** Smoking history and current smoking behavior, smoking cessation history and aids used, Fagerström Test for Cigarette Dependence (FTCD), future plans smoking.

Variable	Total	Dual User	Switcher	Statistic	*p*-Value
Age start smoking ^a^	15.03 (2.61)	14.70 (1.58)	15.10 (2.79)	*t*(213) = −0.88	0.38
Product used before e-cig use					0.45 ^b^
Filtered cigarettes	52	60	50		
Cigarettes without filter	0	0	1		
Rolling tobacco	38	30	39		
Cigars	1	3	1		
Different products	9	8	9		
How often smoked before e-cig use					1 ^b^
Daily	98	100	98		
Multiple days per week, not daily	1	0	2		
Multiple times per month	0	0	1		
Mean CPD before e-cig use ^a^	22.24 (12.34)	22.33 (13.01)	22.23 (12.22)	*t*(213) = 0.05	0.96
Tried to quit smoking?					0.27 ^b^
Yes	88	83	90		
No	12	18	10		
*Mean quit attempts ^a^*	*5.07 (4.62)*	*7.36 (7.13)*	*4.59 (3.75)*	*t(188) = 3.21*	*<0.001*
Longest period quitted smoking (months) ^a^	12.98 (17.14)	9.15 (14.27)	13.79 (17.62)	*t*(188) = −1.42	0.16
*Time since last cigarette smoked*					*<0.001 ^b^*
One day or less	13	68	1		
Between a day and a week	5	20	1		
Between a week and a month	5	10	4		
Between a month and a year	33	3	39		
More than one year	45	0	55		
Cessation aids used quitting smoking					
NRT	52	56	55	χ^2^ (1) = 0.79	0.37
Medication	24	33	32	χ^2^ (1) = 1.53	0.22
E-cig	15	10	11		0.54 ^b^
Counseling	6	19	17	χ^2^ (1) = 3.79	0.05
Willpower (no aid)	58	61	60	χ^2^ (1) = 0.60	0.44
Alternative (laser, hypnotherapy, …)	6	9	8		0.74 ^b^
Cessation aids used longest period quitted smoking					
NRT	12	5	6	χ^2^ (1) = 1.82	0.18
Medication	9	8	8	χ^2^ (1) = 0.02	0.89
E-cig	61	71	69	χ^2^ (1) = 2.69	0.10
Counseling	3	3	3	χ^2^ (1) = 0.01 ^b^	1
Willpower (no aid)	27	18	20	χ^2^ (1) = 0.79	0.38
Alternative (laser, hypnotherapy, …)	0	3	2	χ^2^ (1) = 0.93	1
*Product use now*					*<0.001 ^b^*
Nothing	81	0	100		
Filtered cigarettes	15	83	0		
Rolling tobacco	2	13	0		
Cigars	0	3	0		
Cannabis	0	3	0		
*How often smoking now*				*χ^2^ (3) = 194.06*	*<0.001*
Never	81	0	100		
Daily	9	50	0		
Multiple days per week, not daily	6	30	0		
Multiple times per month	4	20	0		
Mean CPD now ^a^	/	3.92 (5.48)	/	/	/
FTCD-score ^a^	/	3.35 (2.41)	/	/	/
*Future plans regarding smoking*					*<0.001 ^b^*
Do not want to quit completely	3	15	0		
Thinking about quitting, not in next 6 months	3	15	0		
Thinking about quitting, in next 6 months	6	33	0		
Thinking about quitting, in next month	6	33	0		
Thinking about quitting, in next week	1	5	0		
Already quit smoking completely	81	0	100.00		

*Note*: all percentages (rounded to the nearest integer) except for ^a^, ^a^ are *M* (*SD*). ^b^ More than 20% of the expected frequencies less than 5,, Fisher’s exact test was used. *p*-values in *Italic* are significant with α = 0.05.

**Table 3:** E-cig FTCD, current e-cig use, future plans e-cig/nicotine use, and reasons to start and to continue vaping.

Variable	Total	Dual User	Switcher	Statistic	*p*-Value
E-cig FTCD-score ^a^	9.84 (0.51)	9.80 (0.56)	9.85 (0.49)	*t*(213) = −0.58	0.56
Started with e-cigs (months) ^a^	21.81 (19.11)	17.30 (18.37)	22.84 (19.18)	*t*(213) = −1.66	0.10
E-cig type				χ^2^ (2) = 2.80	0.25
Upgrade needed	6	10	5		
Mixed category	25	30	24		
Up-to-date	69	60	71		
Nicotine level					0.75 ^b^
0 mg/mL	11	10	11		
4–8 mg/mL	63	58	64		
9–16 mg/mL	23	30	22		
17–24 mg/mL	3	3	3		
Flavor regular use				χ^2^ (1) = 1.61	0.21
Tobacco	27	35	25		
Other	73	65	75		
Flavor at e-cig initiation				χ^2^ (1) = 2.66	0.26
Tobacco	68	73	67		
Other	32	28	33		
PG/VG proportions				χ^2^ (2) = 3.68	0.16
Dominant PG	27	37	25		
50/50	37	39	37		
Dominant VG	36	24	39		
How often vaping					0.34 ^b^
Daily	99	98	99		
Multiple days per week, not daily	1	3	1		
Puffs per day				χ^2^ (3) = 5.71	0.13
100 puffs or less	16	25	14		
101–200 puffs	34	23	37		
201–300 puffs	28	35	27		
301 puffs or more	22	18	23		
*Mean amount of liquid used (mL/week)*	*38.11 (26.36)*	*23.65 (15.48)*	*41.42 (27.24)*	*t(213) = −3.98*	*<0.001*
Future plans regarding vaping				χ^2^ (2) = 0.05	0.97
Continue to use as now	67	65	67		
Try to cut back	27	28	26		
Try to cut completely	7	8	7		
Future plans regarding nicotine level				χ^2^ (2) = 4.07	0.13
Continue same concentration as now	27	17	24		
Try to cut back	32	44	29		
Switching to zero nicotine	41	39	42		
Reasons to start vaping					
Smoking is unhealthy	4.41 (1.02)	4.43 (0.87)	4.41 (1.05)	*t*(213) = 0.11	0.91
More advantages than other cessation aids	4.37 (1.00)	4.48 (0.72)	4.34 (1.05)	*t*(213) = 0.76	0.45
To quit smoking completely	4.10 (1.33)	3.85 (1.19)	4.16 (1.36)	*t*(213) = −1.33	0.19
Out of curiosity	3.38 (1.40)	3.15 (1.33)	3.43 (1.41)	*t*(213) = −1.16	0.25
Smoking tobacco cigarettes is too expensive	3.28 (1.38)	3.63 (1.44)	3.20 (1.36)	*t*(213) = 1.76	0.08
Smoking bothered other people	2.58 (1.36)	2.45 (1.41)	2.61 (1.36)	*t*(213) = −0.67	0.50
To vape were smoking is prohibited	2.46 (1.32)	2.88 (1.47)	2.36 (1.27)	*t*(213) = 2.24	0.03
*To reduce smoking*	*2.34 (1.40)*	*3.18 (1.36)*	*2.15 (1.34)*	*t(213) = 4.33*	*<0.001*
People around me are also vaping	2.17 (1.67)	2.48 (1.24)	2.10 (1.55)	*t*(213) = 1.83	0.07
Reasons to continue vaping					
Smoking is unhealthy	4.63 (0.78)	4.53 (0.85)	4.66 (0.76)	*t*(213) = −0.97	0.33
More advantages than other cessation aids	4.58 (0.79)	4.60 (0.74)	4.57 (0.80)	*t*(213) = 0.21	0.84
It is pleasant	4.56 (0.62)	4.48 (0.55)	4.58 (0.64)	*t*(213) = −0.99	0.32
Smoking tobacco cigarettes is too expensive	3.48 (1.27)	3.68 (1.31)	3.44 (1.26)	*t*(213) = 1.06	0.29
Smoking bothered other people	3.39 (1.21)	3.23 (1.27)	3.42 (1.91)	*t*(213) = −0.94	0.35
To vape were smoking is prohibited	2.65 (1.26)	2.95 (1.50)	2.58 (1.20)	*t*(213) = 1.69	0.09
People around me are also vaping	2.27 (1.12)	2.33 (1.21)	2.26 (1.10)	*t*(213) = 0.32	0.75
It helps me to smoke less ^a^	/	4.65 (0.77)	/	/	/
To quit smoking completely in time ^a^	/	4.30 (0.99)	/	/	/
To reduce smoking ^a^	/	2.28 (1.50)	/	/	/
To prevent relapsing to smoking ^a^	/	/	4.24 (1.00)	/	/

*Note*: all percentages (rounded to the nearest integer) except for ^a^ and ^c^, ^a^ are *M* (*SD*), ^c^ are *n*. ^b^ More than 20% of the expected frequencies less than 5, Fisher’s exact test was used. *p*-values in *Italic* are significant with α = 0.05 or adjusted α of 0.006 and 0.007 for “Reasons to start vaping” and “Reasons to continue vaping”, respectively.

**Table 4:** Risk perception, e-cig efficacy, practical problems, social component, negative and positive consequences, ‘addiction’ to cigarettes/e-cigs/nicotine.

Variable	Total	Dual User	Switcher	Statistic	*p*-Value
Risk perception	1.86 (0.62)	1.83 (0.66)	1.86 (0.61)	*t*(213) = −0.37	0.72
The faster I quit vaping, the better	2.56 (1.14)	2.63 (1.30)	2.54 (1.11)	*t*(213) = 0.41	0.68
Concerns regarding long term health effects e-cig	2.31 (1.01)	2.13 (1.02)	2.35 (1.01)	*t*(213) = −1.27	0.21
Concerns regarding long term e-cig use	1.95 (0.87)	1.85 (0.92)	1.98 (0.86)	*t*(213) = −0.83	0.41
Fear problems with heart and blood vessels	1.69 (0.74)	1.73 (0.85)	1.69 (0.71)	*t*(213) = 0.30	0.76
Fear breathing problems due to e-cig use	1.65 (0.71)	1.58 (0.64)	1.67 (0.72)	*t*(213) = 0.76	0.45
Fear for lung cancer	1.59 (0.74)	1.60 (0.87)	1.59 (0.71)	*t*(213) = 0.09	0.93
E-cig is as harmful as a cigarette	1.25 (0.50)	1.28 (0.51)	1.24 (0.50)	*t*(213) = 0.40	0.69
*E-cig efficacy*	*4.30 (0.45)*	*3.97 (0.56)*	*4.37 (0.40)*	*t(213) = −5.18*	*<0.001*
*Less desire for cigarettes*	*4.89 (0.37)*	*4.60 (0.67)*	*4.96 (0.20)*	*t(213) = −6.08*	*<0.001*
Decreased smoking	4.81 (0.69)	4.88 (0.34)	4.79 (0.75)	*t*(213) = 0.67	0.50
*After vaping I do not immediately smoke*	*4.73 (0.78)*	*3.90 (1.34)*	*4.93 (0.39)*	*t(213) = −8.74*	*<0.001*
*No need for cigarette after vaping for a while*	*4.70 (0.77)*	*4.25 (0.84)*	*4.80 (0.72)*	*t(213) = −4.23*	*<0.001*
Less desire for nicotine	4.27 (0.92)	4.18 (0.93)	4.30 (0.92)	*t*(213) = −0.75	0.45
*Throat hit is comparable*	*3.47 (1.08)*	*3.00 (1.11)*	*3.57 (1.04)*	*t(213) = −3.09*	*0.002*
Act vaping is comparable with smoking	3.20 (1.24)	3.00 (1.34)	3.25 (1.21)	*t*(213) = −1.16	0.25
*Practical problems*	*1.87 (0.71)*	*2.16 (0.74)*	*1.80 (0.69)*	*t(213) = 2.94*	*0.004*
Purchasing liquids is difficult	1.99 (1.26)	2.18 (1.28)	1.94 (1.25)	*t*(213) = 1.06	0.29
*Battery empty at inconvenient moments*	*1.97 (1.00)*	*2.48 (1.06)*	*1.85 (0.95)*	*t(213) = 3.65*	*<0.001*
*Inconvenient to carry material of e-cig*	*1.92 (0.98)*	*2.40 (1.19)*	*1.81 (0.89)*	*t(213) = 3.53*	*0.001*
Purchasing e-cig is difficult	1.92 (1.83)	2.00 (1.13)	1.90 (1.20)	*t*(213) = 0.47	0.64
Difficult to not forget anything for using e-cig	1.89 (0.96)	2.25 (1.08)	1.81 (0.91)	*t*(213) = 2.68	0.008
Purchasing coils and other parts is difficult	1.84 (1.14)	2.00 (1.16)	1.81 (1.13)	*t*(213) = 0.98	0.33
Malfunction e-cig	1.57 (0.79)	1.85 (0.98)	1.15 (0.73)	*t*(213) = 2.49	0.01
Social component	3.33 (0.65)	3.41 (0.75)	3.31 (0.62)	*t*(213) = 0.93	0.35
Environment obligates me to vape outside	4.00 (1.13)	3.95 (1.32)	4.01 (1.08)	*t*(213) = −0.28	0.78
Environment still sees me as addicted	3.65 (0.91)	3.90 (0.98)	3.59 (0.89)	*t*(213) = 1.93	0.06
Others see vapor as harmful as cigarette smoke	3.64 (1.06)	3.65 (1.15)	3.63 (1.04)	*t*(213) = 0.09	0.93
I feel obligated to stand outside with smokers	3.35 (1.30)	3.38 (1.44)	3.35 (1.27)	*t*(213) = 0.12	0.91
When vaping, others still consider me as a smoker	3.34 (1.17)	3.58 (1.08)	3.29 (1.83)	*t*(213) = 1.42	0.16
Environment sees switch to e-cig as not positive	1.98 (0.83)	2.03 (0.92)	1.97 (0.81)	*t*(213) = 0.37	0.71
*Negative consequences*	*1.52 (0.36)*	*1.67 (0.42)*	*1.49 (0.34)*	*t(213) = 2.92*	*0.004*
More technical problems	2.75 (1.07)	2.78 (1.03)	2.75 (1.09)	*t*(213) = 0.14	0.89
Dry mouth	2.40 (1.00)	2.60 (1.03)	2.35 (0.99)	*t*(213) = 1.44	0.15
Dry throat	2.10 (0.96)	2.30 (1.04)	2.05 (0.94)	*t*(213) = 1.48	0.14
Increased weight	1.64 (1.01)	1.50 (0.85)	1.67 (1.04)	*t*(213) = −0.99	0.33
*Coughing*	*1.52 (0.70)*	*1.83 (0.87)*	*1.45 (0.64)*	*t(213) = 3.09*	*0.002*
*Bad taste when vaping*	*1.51 (0.69)*	*1.88 (0.85)*	*1.42 (0.62)*	*t(213) = 3.86*	*<0.001*
Sore throat	1.39 (0.62)	1.63 (0.81)	1.33 (0.55)	*t*(213) = 2.77	0.006
Worrying about health	1.37 (0.61)	1.40 (0.67)	1.37 (0.60)	*t*(213) = 0.32	0.75
Unpleasant sensation in throat	1.33 (0.58)	1.48 (0.72)	1.30 (0.54)	*t*(213) = 1.70	0.09
*Bad physical condition*	*1.30 (0.63)*	*1.60 (0.87)*	*1.23 (0.54)*	*t(213) = 3.38*	*0.001*
*Sleeping problems*	*1.28 (0.70)*	*1.60 (0.96)*	*1.21 (0.60)*	*t(213) = 3.26*	*0.001*
Headache	1.27 (0.54)	1.33 (0.57)	1.26 (0.53)	*t*(213) = 0.72	0.47
Increased heart rate or palpitations	1.24 (0.52)	1.40 (0.63)	1.20 (0.48)	*t*(213) = 2.23	0.03
Unpleasant odors when using	1.23 (0.50)	1.20 (0.41)	1.23 (0.52)	*t*(213) = −0.39	0.70
Bad taste	1.22 (0.55)	1.28 (0.60)	1.21 (0.54)	*t*(213) = 0.72	0.47
Breathing problems	1.21 (0.47)	1.40 (0.63)	1.17 (0.42)	*t*(213) = 2.79	0.006
Bad smell	1.13 (0.37)	1.23 (0.53)	1.11 (0.32)	*t*(213) = 1.72	0.09
*Positive consequences*	*4.20 (0.49)*	*3.84 (0.56)*	*4.28 (0.43)*	*t(213) = −5.45*	*<0.001*
*Craving for cigarette is reduced*	*4.87 (0.38)*	*4.63 (0.59)*	*4.93 (0.28)*	*t(213) = −4.78*	*<0.001*
Could decrease smoking	4.71 (0.71)	4.78 (0.42)	4.69 (0.76)	*t*(213) = 0.67	0.50
*Could quit smoking*	*4.59 (0.90)*	*3.10 (0.98)*	*4.93 (0.39)*	*t(213) = −19.06*	*<0.001*
*Smell/taste cigarette is less pleasant*	*4.54 (0.91)*	*4.13 (1.14)*	*4.64 (0.82)*	*t(213) = −3.32*	*0.001*
Fresher breath	4.49 (0.73)	4.33 (0.69)	4.53 (0.73)	*t*(213) = −1.58	0.12
More pleasure in vaping than in smoking	4.38 (0.90)	4.10 (1.08)	4.45 (0.84)	*t*(213) = −2.22	0.03
*Fitness and health are improved*	*4.38 (0.82)*	*4.00 (1.01)*	*4.47 (0.75)*	*t(213) = −3.33*	*0.001*
Less coughing	4.36 (0.98)	4.00 (1.13)	4.44 (0.92)	*t*(213) = −2.61	0.01
*Better breathing*	*4.31 (0.83)*	*3.88 (0.91)*	*4.41 (0.77)*	*t(213) = −3.78*	*<0.001*
*Improved sense of smell*	*4.24 (0.88)*	*3.68 (0.97)*	*4.37 (0.81)*	*t(213) = −4.68*	*<0.001*
*Improved taste*	*4.24 (0.84)*	*3.75 (0.87)*	*4.35 (0.80)*	*t(213) = −4.26*	*<0.001*
Less disturbing for other people	3.84 (1.06)	3.65 (1.25)	3.89 (1.01)	*t*(213) = −1.28	0.20
More often in a good mood	3.62 (1.04)	3.8 (1.09)	3.65 (1.03)	*t*(213) = −0.97	0.34
Improved sleep quality	3.58 (1.01)	3.35 (1.00)	3.63 (1.01)	*t*(213) = −1.58	0.12
Possible to vape in several places	3.57 (1.29)	3.68 (1.42)	3.55 (1.26)	*t*(213) = 0.56	0.58
*Improved appetite*	*3.50 (0.98)*	*3.00 (0.93)*	*3.62 (0.95)*	*t(213) = −3.72*	*<0.001*
*‘Addiction’ to cigarettes*	*1.76 (1.06)*	*3.28 (0.95)*	*1.41 (0.72)*	*t(213) = 13.85*	*<0.001*
*I cannot resist the act of smoking*	*1.92 (1.32)*	*3.38 (1.08)*	*1.58 (1.13)*	*t(213) = 9.15*	*<0.001*
*Addicted to cigarette at this moment*	*1.60 (1.06)*	*3.18 (1.08)*	*1.23 (0.64)*	*t(213) = 14.93*	*<0.001*
I consider my addiction to cigarettes as problematic ^a^	/	3.53 (1.33)	/	/	/
‘Addiction’ to e-cigs	3.53 (0.93)	3.28 (1.09)	3.59 (0.88)	*t*(213) = −1.98	0.05
Addicted to e-cig at this moment	3.55 (0.97)	3.28 (1.15)	3.62 (0.92)	*t*(213) = −2.02	0.05
I cannot resist the act of vaping	3.52 (1.02)	3.28 (1.11)	3.57 (0.99)	*t*(213) = −1.67	0.10
I consider my addiction to e-cigs as problematic ^a^	1.83 (0.82)	2.13 (0.92)	1.77 (0.79)	*t*(135) = 1.94	0.06
‘Addiction’ to nicotine	2.66 (0.94)	2.93 (0.99)	2.60 (0.92)	*t*(213) = 1.97	0.05
I am more addicted to nicotine since vaping	2.00 (1.09)	2.13 (1.20)	1.97 (1.06)	*t*(213) = 0.80	0.42
*I feel addicted to nicotine*	*3.33 (1.18)*	*3.73 (1.18)*	*3.23 (1.16)*	*t(213) = 2.10*	*0.02*

*Note*: all means of a 5-point scale, ( ) are *SD*’s. ^a^ are not included in overall statement. *p*-values in *Italic* are significant with α = 0.05 (average scale scores) or Bonferroni adjusted α (individual items of scales).
